# Anaplasty

**Published:** 1845-08-01

**Authors:** F. H. Hamilton

**Affiliations:** Professor of Surgery in Geneva Medical College


### Anaplasty.

Communicated for the Buffalo Medical Journal, by F. H. HAMILTON, M. D., Professor of Surgery in Geneva Medical College.

Anaplasty, or the art of reconstructing mutilated and destroyed parts, although in some way very ancient, has but recently attained its greatest perfection. Perhaps, indeed, we should not be allowed to speak thus boastingly of the improvements of the present age, when we remember that Dujardin has recorded of Esculapius that he replaced and made to unite, the head of a decapitated woman! and that Sedillot has affirmed of a soldier that "decapiti par un enemi, fut gueri par un paysan," unfortunately, however, the peasant placed the head wrong side before, and rendered him of no farther service to the army except as a rear-guard; and since by Rabelais we are assured that Epistemon having lost his head, Panurgus had the skill to make it unite again "veine contre veine, nerf contre nerf, spondyle contre spondyle," and thus to affect a most perfect cure, "except that his voice remained hoarse, and that he had a dry cough, which he never was relieved of but by the aid of liquor"! These are indeed marvelous cases, and might go far to show that Anaplastic Surgery had rather retrograded since the days of Esculapius. But if the present age cannot record any one operation so wonderful as those just related, it may, at least, boast that the art of "restitution" has been greatly extended.

Tagliacozzi was among the first who (after Esculapius!) attempted to restore lost organs, by whom the operation was confined, however, to the formation of new noses; and it is only within a few years that it has been made to apply to the restoration of more or less of almost every portion of the external superficies of the body. To the art in general the term "Anaplasty" or "Autoplasty" is now applied. While the specific forms of Anaplasty are designated by appropriate and distinct appellations.—Anaplasty has been performed in various ways.

First—by removing a portion of living texture from one animal to another and compelling it to unite ; as in the engrafting of the spur of one cock upon the crest of another : the transplanting and uniting the tooth of one person to the socket of another, and even the tooth of a lamb to the socket of a young lady; (related by Mr. Twiss of Kerry Ireland.) It is said even that in this way a man has been robbed of his nose to supply the defective organ upon the face of another.

Second—by supplying the deficiency from textures furnished by the same body, but wholly disconnected from the part to be restored. It is thus that M. Dutrochet relates an operation for restoration of the nose, made by an Indian, who selected a place "de la fesse" which he tumefied by blows with a slipper, and from this integument thus rendered active and succulent, cut out a piece and restored the nose.

Third. *Italian process*—by obtaining integuments, &c., from parts naturally remote, but which by position can be brought into contact with the part to be restored. Thus Tagliacozzi brought the arm in contact with the face and secured it there until a flap *partially* detached from the arm had been made to unite with the face.

Fourth—by cutting from a part *naturally in close proximity* and uniting the flaps to the mutilated organ, before it has been completely separated from its original seat, (as in the third method.) Of this method there exists two principal and many subordinate modifications.

*First*—the Indian method, which consists in forming the flaps with a narrow pedicle and applying it to its new seat by reversion, or twisting.

*Second*—The French method, which consists in forming the flaps with a wider pedicle or base and stretching it into its new bed, without any torsion.

One or the other of the two latter methods, more or less varied, are now generally preferred.

In the two first cases below reported it will be seen that I selected the French method, or the method "par glissement du lambeau," as being always preferable to the Indian, wherever the cellular texture and integuments are sufficiently loose to allow the requisite extension ; since it is less liable to loose its vascularity and to slough than when tortion is practiced.

*Blepharoplasty.*—Albert P. Heth, of Seneca Falls, aged 18 years. In consequence of small pox about one year since, almost the whole integuments of the face became permanently thickened and indurated, and covered with pits and seams. The only portions of the face which are not thus thickened, &c., are the upper eyelids and a crescent shaped portion of integument below the eyes, of one inch and a half in breadth, by one inch in depth at the widest part of the crescent. Both of the under lids are inverted and drawn down in the center ; exposing upon each cheek a red and spongy texture nearly in the form of the letter V, with its base upwards ; which base corresponds in breadth with the space between the outer and inner canthus ; and its apex extending downwards about ¾ of an inch, or nearly to the lower margin of the crescent.

In this case no integument could be borrowed from the adjacent tissues to supply the lids, as there was none in a fit condition. To make the operation from the arm was not to be preferred. I therefore selected a mode which, although novel has proved successful.

*Operation*, January 8, 1845. First—I made an incision in the form of the letter V, with its base upwards, corresponding, of course, to the shape of the deformity; but commencing about three lines from each canthus, so as to leave in all, half an inch of tarsal margin. These incisions being intended to scarify and expose a clean surface for adhesion, were carried upon the sound integument, near its border, and were made to extend below to the indurated integument before they met, the thread like border thus intercepted being immediately dissected off.

Second—the knife was passed around the whole convex edge of the crescent; along the line between the sound and indurated integument.

Third—the two flaps formed between the outer sides of the V incision, and the crescentic incision, which incisions intersected each other at the apex of the V, were then dissected up to the margin of the tarsus.

Fourth—the opposite flaps were now "slid" up and two small sutures made to unite them; the first being passed through the tarsal border, and the second a little below. The force required to bring the tarsal margins together, although half an inch had been removed, was very inconsiderable.

Fifth—a very slight compress sustained by one or two small strips of adhesive plaster completed the dressings.

Dec. 13—removed the sutures — union complete—continue light dressing.

Dec. 16 — the eyelids close perfectly. The space, below the flaps which were carried up, is beginning to cicatrize by formation of new cutis. Applied a small compress under lower lid to prevent as far as possible its depression during the cicatrization.

On the 16th Jan., I operated in the same manner upon the opposite lid and with equal success. The patient returned home on the 18th (contrary to my wish.) The cicatrization of the raw surface had already made considerable progress in the first eye operated upon, and the lids still retained their position. I however, warned the lad that against a *partial* return of the deformity, he had no guarantee. Says Velpeau, "whichsoever method we take, the eyelid the best reconstructed, rarely fails to become again deformed" (de se de'former de noveau.")—By which he explains himself to mean that *some degree* of deformity, generally vastly less than before, returns.

*Cheiloplasty and Relief of Permanent Closure of Jaws.* — Miss ———, of Yates Co., aged about 18 years—has a permanent closure of jaws, with deformity of mouth, in consequence of excessive salivation, ulceration and sloughing, during infancy. Mouth can now be separated only 3 or 4 lines: most of teeth of left side removed by the disease: left angle of the mouth very much elongated and drawn down.

Dec. 5th. 1844.—*Operation.* First—the integument was carefully dissected from left angle of mouth to the distance of eight lines upon each lip, for the purpose of coapting and restoring by adhesion an equal length to the mouth on both sides of the mesian line. Second—the firm callous adhesions which held the jaws together, were divided with a probe pointed bistoury, cutting horizontally, also upwards and downwards, outside of the alveoli, until all the contractions were severed. The jaws were now easily separated to twice the former distance; but no violence was used, as it was proposed to accomplish this gradually, so that the articulations should not suffer. The hemorrhage, at first considerable, soon ceased spontaneously. Third, a piece of wood properly carved and made smooth, was now introduced outside of the alveoli, to prevent renewed adhesions between the alveoli and cheek. Fourth, the abraded lips (first incision) were brought in contact and retained by 3 sutures, over which adhesive straps, light compresses and rollers completed the dressing. Water gruel, rice, &c., directed as diet.

10th. A small apparatus placed between the front teeth (which had previously been kept asunder by wedges) for the purpose of producing a gradual and steady separation. The piece of carved wood also removed and lint substituted. The wood has answered an excellent purpose in thus far preventing adhesion.

The patient returned home in about three weeks from the date of the operation, the wounds having entirely healed, and the jaws separating easily an inch. She was advised to continue to wear the apparatus between her teeth except when eating, as it does not interfere with the speech, and gradually to increase the separation.

The apparatus of which I speak consisted of a male and female screw, each 5 lines in length, supporting at their opposite extremities two silver caps which were fitted to an upper and lower front tooth. The two portions of the screw being then closed into one another and the caps adjusted, they were made to separate by introducing a small steel lever through a fenestra in the male screw and turning.

*Rhino-plasty.*—Elias Beardsley, of Catherine, Chemung Co. aged 11 years, in consequence of an extensive lacerated wound, received when 4 years old, had lost a portion of right ala of nose, accompanied with a closure of the nostril immediately below the point of deficiency. Two unsuccessful attempts to restore the nose had been made, one in 1840 and the other in 1842.

*Operation*, Nov. 30, 1844. The natural channel having been previously opened by a trocar, the operation for restoration of the side of the nose was commenced. First, the edges of the deficiency were freely excised with a scalpel. Second, two parallel incisions were made, commencing at the upper aud lower outer angles of the opening, and extending outwards and downwards one inch and a half: the intercepted portion of integument was then dissected from the subjacent tissues and permitted to continue attached to the remaining integuments of the face only by its base. The operation was now suspended a few moments to allow the hermorrhage entirely to cease. Third, while an assistant supported the cheek, the flaps of integument were drawn forcible up and secured accurately by six delicate sutures to the abraded edges. Fourth, the assistant still continuing to sustain the cheek, several strips of adhesive plaster, broadest upon the face, were laid obliquely from the angle of the jaw upwards and forwards across the bridge of the nose, terminating upon the forehead. These served effectually to prevent all strain upon the sutures. Fifth, a light dressing consisting of a small linen rag covered with cerate.

Dec. 4th. The straps have been carefully renewed each day. To-day removed 3 of the sutures.

Dec. 5th. Removed the remainder of the sutures; the adhesion of the flap is complete at every point; continued adhesive plasters and a simple dressing.

Dec. 11. To-day I separated the base of the flaps from the face (which continued to pull forcibly upon the nose whenever the adhesive plasters were removed,) introducing a long narrow pledget of lint to prevent a reunion ; adhesive straps discontinued

The lad returned home about the 20th entirely cured.

